# Detection of SARS-CoV-2 Neutralizing Antibodies in Vaccinated Pregnant Women and Neonates by Using a Lateral Flow Immunoassay Coupled with a Spectrum-Based Reader

**DOI:** 10.3390/bios12100891

**Published:** 2022-10-18

**Authors:** Wei-Chun Chen, Yen-Pin Lin, Chao-Min Cheng, Ching-Fen Shen, Chang-Wei Li, Yu-Kuo Wang, Ting-Ying Shih, Chitsung Hong, Ting-Chang Chang, Ching-Ju Shen

**Affiliations:** 1 Institute of Biomedical Engineering, National Tsing Hua University, Hsinchu 300, Taiwan; 2 Division of Gynecologic Oncology, Department of Obstetrics and Gynecology, Chang Gung Memorial Hospital at Linkou, College of Medicine, Chang Gung University, Taoyuan 333, Taiwan; 3 Department of Obstetrics and Gynecology, New Taipei City Municipal Tucheng Hospital, New Taipei City 236, Taiwan; 4 Department of Pediatrics, National Cheng Kung University Hospital, College of Medicine, National Cheng Kung University, Tainan 701, Taiwan; 5 AllBio Life Inc., Taichung 408, Taiwan; 6 Spectrochip Inc., Hsinchu 302, Taiwan; 7 Department of Obstetrics and Gynecology, Kaohsiung Medical University Hospital, Kaohsiung Medical University, Kaohsiung 807, Taiwan

**Keywords:** COVID-19 vaccine, wild-type SARS-CoV-2, Delta-type SARS-CoV-2, Omicron-type SARS-CoV-2, S1RBD IgG, neutralizing antibodies, lateral flow immunoassay

## Abstract

The focus of this study was to investigate the detection of neutralizing antibodies (Nabs) in maternal serum and cord blood as the targeted samples by employing a lateral flow immunoassay combined with a spectrum reader (LFI-SR) and the correlation of Nab protection against different types of SARS-CoV-2. We enrolled 20 pregnant women who were vaccinated with the Moderna (mRNA-1273) vaccine during pregnancy and collected 40 samples during delivery. We used an LFI-SR for the level of spike protein receptor binding domain antibody (SRBD IgG) as Nabs and examined the correlation of the SRBD IgG concentration and Nab inhibition rates (NabIR) via enzyme-linked immunosorbent assays (ELISA). The LFI-SR had high confidence for the SRBD IgG level (*p* < 0.0001). Better NabIR were found in wild-type SARS-CoV-2 (WT) compared to Delta-type (DT) and Omicron-type (OT). Women with two-dose vaccinations demonstrated greater NabIR than those with a single dose. The cut-off value of the SRBD IgG level by the LFI-SR for NabIR to DT (≥30%; ≥70%) was 60.15 and 150.21 ng/mL for mothers (both *p* = 0.005), and 156.31 (*p* = 0.011) and 230.20 ng/mL (*p* = 0.006) for babies, respectively. An additional vaccine booster may be considered for those mothers with SRBD IgG levels < 60.15 ng/mL, and close protection should be given for those neonates with SRBD IgG levels < 150.21 ng/mL, since there is no available vaccine for them.

## 1. Introduction

Coronavirus disease 2019 (COVID-19) is an infectious disease caused by severe acute respiratory syndrome coronavirus 2 (SARS-CoV-2) [1]. SARS-CoV-2 has a high transmission rate which led to the rapid spreading of the disease and increasing numbers of infected people [2]. The advent of vaccines for COVID-19 has been a solution to the spread of the disease [3,4]. The vaccine can also prevent severe illness and may be considered especially useful for at-risk populations, which include not only those who may be vulnerable due to existing physical conditions, but those with vulnerable cohorts, such as pregnant women and healthcare providers [5,6].

Pregnant women may be at higher risk of developing severe illness while infected with COVID-19 [7,8]. A systemic review by Pettirosso et al., reported that 18% of pregnant women experienced severe illness and 5% experienced critical disease states. These rates were higher than those found in the general population, i.e., 14% with severe morbidity and 5% with critical illness [9]. Another large study examining pregnancy-associated conditions included a total of 342,080 women, 3527 of whom had confirmed cases of COVID-19 at the time of birth. The associated risks for pregnant women infected with SARS-CoV-2 included preterm birth, pre-eclampsia, and emergency Cesarean delivery [10]. Additionally, neonatal COVID-19 infection may increase rates of fetal death, neonatal adverse outcomes, and prolonged neonatal hospitalization after birth [10,11]. Previous reports examining pregnant women receiving vaccines, such as an influenza vaccine, meningococcal vaccine, and Tetanus toxoid vaccine, have shown that the mother can produce maternal antibodies and provide them to the fetus through intrauterine, transplacental transfer without harm to the mother or the fetus [12]. Additionally, previous studies have indicated that the fetus can acquire antibodies from infected mothers via the transplacental route [13]. In our previous research, we found that the transplacental transmission of maternal neutralizing antibodies (Nabs) to SARS-CoV-2 could also be detected and could provide acquired protection and innate immunity for both the fetus and neonate [14]. However, variants of SARS-CoV-2 may decrease the efficacy of vaccines and lead to breakthrough COVID-19 infections [15]. The measurement of Nab concentration or Nab inhibition rates for SARS-CoV-2 may be used to determine potential vaccine efficacy. Furthermore, antibodies to the spike 1 protein receptor binding domain (S1RBD IgG) demonstrated the ability to neutralize virus-to-host cell attachment, thus, SRBD IgG detection may be construed as Nab detection [16,17]. Our previous research also demonstrated that those receiving only one dose of the vaccine had poor Nab protection compared to those with full, two-dose vaccination, and the studied vaccine, Moderna (mRNA-1273), provides lower Nab inhibition rates to Delta variants compared to wild-type (WT) SARS-CoV-2 infection [14].

It is important to provide rapid detection of the Nab level or SRBD IgG concentration in order to determine whether vaccine protection is still sufficient. Lateral flow immunoassays have been developed after the evolution of radio-immunoassays, enzyme immunoassays, and lateral flow technology since the 1960s [18]. Using lateral flow immunoassays for human chorionic gonadotropin (hCG) as a rapid test in pregnancy has been used for years [19]. Nowadays, lateral flow immunoassays have been used for the detection of hormones, biomarkers, toxins, and even antibodies against different viruses, including SARS-CoV-2 [20,21,22]. Since lateral flow immunoassays have the advantages of simple use by providing rapid results, they have been widely used as rapid test tools for point-of-care detection [23,24], but they were hampered by the fact that it was difficult to make quantitative evaluations of the colorimetric results with the naked eye. Our previous research used lateral flow immunoassays in combination with a portable spectrum reader to detect the reflectance spectrum of the colorimetric change on lateral flow strips for quantifying the serum Nab (SRBD IgG) level in COVID-19-infected patients [17].

The aim of this current study was to examine women and their delivered neonates, and compare the serum SRBD IgG level results provided via the use of a lateral flow immunoassay in combination with a spectrum reader to the results provided via a traditional Nab inhibition rate evaluation using enzyme-linked immunosorbent assay (ELISA). The focus of this effort was to then determine the relative performance of a rapid test strip technique for detecting the effectiveness of vaccines against different types of SARS-CoV-2 among these vulnerable cohorts.

## 2. Materials and Methods

### 2.1. Patient Selection

This prospective study was performed at Kaohsiung Medical University Hospital. All participants were enrolled during admission before delivery, and all were confirmed SARS-CoV-2 infection-negative via a nasopharyngeal swab polymerase chain reaction test at the time. Patients with preterm labor were not enrolled in our study. Furthermore, all enrolled participants had singleton pregnancies, 17 of them received the second dose of the Moderna (mRNA-1273) vaccine between the 27th and 38th weeks of gestation, and all were confirmed to not have any COVID-19-related symptoms during pregnancy. Vaccinations were performed during pregnancy, and all participants were ≥20 years of age with no medical history of disease requiring immunosuppressant treatment.

We collected peripheral blood and umbilical cord blood as maternal and fetal blood samples, respectively. Both blood samples were obtained on the day of birth, and the fetal blood samples were collected from the umbilical cord after cord clamping. Furthermore, all associated clinical data, including enrolled age, body mass index (BMI), weeks of vaccination, delivery weeks, and other available information, were obtained from the electronic medical record system. This prospective study was conducted with the approval of an institutional review board (IRB) (IRB number: KMUHIRB-SV(II)-20210087).

All patients in this study had singleton pregnancies without symptoms related to COVID-19, and all were voluntarily vaccinated against COVID-19 during pregnancy. The exclusion criteria were as follows: (1) age < 20 years; (2) COVID-19 vaccination before pregnancy; (3) preterm labor; and (4) disease with immunosuppressant treatment.

### 2.2. Rapid Test Strip by Lateral Flow Immunoassays

**Figure 1:**
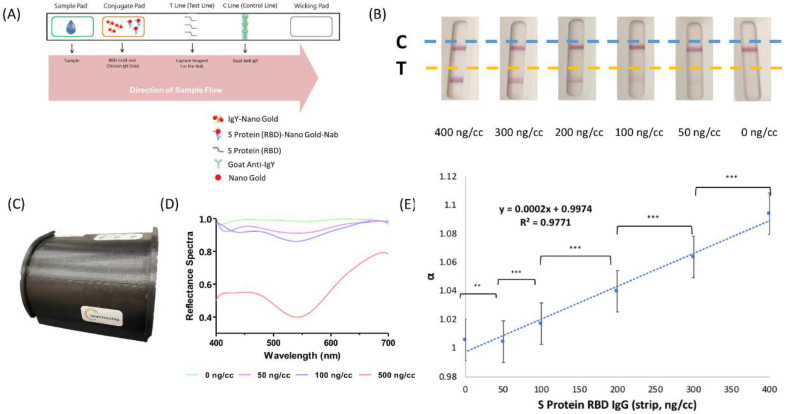
(**A**) The colorimetric change mechanism aspect of the lateral flow immunoassay strip. (**B**) The colorimetric change in a lateral flow rapid test strip for detecting neutralizing antibodies. The control line is shown as a blue dotted line, the test line as a yellow dotted line. (**C**) The spectrum analyzer used in our study. The analyzer can load a lateral flow immunoassay and can be used in combination with a spectrum-based reader. (**D**) The reflectance spectra range of the control line and test line. (**E**) Standard curve of SRBD IgG by a lateral flow immunoassay/spectrum analyzer. SRBD, spike protein receptor binding domain; ** *p* < 0.01; *** *p* < 0.001.

We used a SARS-CoV-2 rapid test strip to detect spike protein receptor binding domain (SRBD) IgG in maternal serum and neonatal cord blood. Here, we tested the SRBD complex instead of S1RBD IgG, since the S1RBD IgG amount in the sample may not be enough to demonstrate significantly visible results. Additionally, S1RBD IgG was used in the Enzyme-linked Immunosorbent Assay (ELISA) mentioned in a later section for precise assessments. The commercialized rapid test strip (AllBio Science, Taichung, Taiwan) employs a lateral flow immunochromatographic methodology with a strip containing spike protein receptor binding domain (SRBD) antigen-coated gold particles. The size of the colloidal gold used to make our lateral flow immunoassay was about 40 nm, and is commercially available from BBI Solution (Cat. No.: EM.GC40). The targeted neutralizing antibodies (Nabs) in serum were detected by a double-antigen sandwich lateral flow immunoassay. As demonstrated in Figure 1A, a rapid test strip primarily comprised a sample well, a conjugated pad, a T line (test line), and a C line (control line).

The serum sample of approximately 25 µL and one drop of buffer (about 40 µL) was dropped into the sample well sequentially, where it was allowed to migrate along the strip via capillary movement. Recombinant viral SARS-CoV-2 protein (SRBD) conjugated with gold colloids was precoated on the conjugated pad to provide a colorimetric presentation. Recombinant SARS-CoV-2 protein (SRBD) was precoated at the test line. If there were Nabs in the serum sample, it conjugated with the SRBD–gold particles that were then captured and formed a complex with the SRBD at the test line, where a visible red line was generated. If, however, there were no Nabs in the serum sample, the SRBD–gold particles were not carried by Nabs, and no red line appeared at the test line region. We also placed the rapid test strip results into the spectrum analyzer to demonstrate the results shown in Figure 1B. The higher the Nab concentration within the serum sample, the greater the color intensity as read by the spectrum analyzer, which provided a quantitative measurement value. Quality control antibody was coated upon the strip at the control line, which provided a resulting red line after sample exposure in order to confirm whether the sample volume was of quality. Regardless of the test line results, there was always a red line at the control line region. In other words, no matter what the test line result is, there should always be a red line found at the control line region to confirm the suitability of the sample and the test strip.

### 2.3. Reflectance Spectral Analysis

As shown in Figure 1C, reflectance spectral analysis was performed with a spectrum analyzer (Taiwan SpectroChip Inc.; Hsinchu, Taiwan FDA: MD (I)-008090 and US FDA: 3017810861) equipped with a rapid test strip cassette for lateral flow immunoassays, and a corresponding spectrum reader was used to detect the spectrum of SARS-CoV-2 Nabs in the serum sample. During examination, a continuous spectrum could be generated from the analyzer, and an optical module of the analyzer was capable of capturing the high-resolution reflectance spectrum of the test line region on the test strip. Further analysis of the optical signal was done by the spectrum reader.

As shown in Figure 1D, the spectrum reader offered high-resolution results up to 3–5 nm across a spectral range as wide as 300–1100 nm. Furthermore, the reader primarily detected the primary reflectance wavelengths as 430 and 600 nm, with a main reference wavelength of 650 nm. The spectrum detection applied white light for scanning. While the red line (test line) was scanned, the light with a spectrum between 430 nm and 600 nm was absorbed by colloidal gold, so the reflected light was the remaining light that was not absorbed. The ratio of the minimum reflectance to the spectrum at the reference wavelength can be defined as the α value:

α = Reflectance (650 nm)/Reflectance (the minimum value in the range of 430–600 nm)

Since the spectrum at 650 nm was not absorbed by colloidal gold, the reflectance value of the red line for 650 nm was about 1. From this formula, if the sample had a higher SRBD IgG concentration, there would be a more obvious red color line in the test line region, and the reflectance value of the red line with a spectrum at 430–600 nm would be lower, and we would obtain a higher α value. Therefore, by the same principle, if there is a lower SRBD IgG concentration in the sample, the reflectance value at 430–600 nm will be higher, and the detected α value will be lower. In other words, the higher the α value, the stronger color intensity of the test line in the strip that indicated higher SRBD IgG concentration.

### 2.4. S1 Receptor Binding Domain IgG Antibody Detection by Enzyme-Linked Immunosorbent Assay (ELISA)

We used sandwich ELISA to detect S1RBD IgG antibody to validate the rapid test strip performance. The ELISA-based detection of S1RBD IgG antibody was performed using a commercialized human IgG ELISA kit (RayBiotech, Peachtree Corners, GA, USA; Cat.: IEQ-CoVS1RBD-IgG-1) under the manufacturer’s instructions. The samples and positive control were added to a 96-well plate coated with S1RBD protein after dilution. After 1 h of incubation at room temperature, the wells were washed and biotinylated anti-human IgG antibody was added. After 30 min of incubation, the wells were washed again and HRP-conjugated streptavidin was added. The wells were washed again after another 30 min of incubation, and TMB substrate solution, as well as stop solution, were subsequently added in the dark to see whether or not there was a colorimetric change from blue to yellow. Color intensity was measured at 450 nm via an OD reader (Molecular Devices, San Jose, CA, USA).

Linear regression was also used to determine the performance of the lateral flow immunoassay test strip/spectrum analyzer using a standard concentration gradient to determine the rapid test strip SRBD IgG value. The linear regression was used to validate the performance of the test strip and obtain a trend line equation. Furthermore, the limit of detection (LOD) and the limit of quantification (LOQ) were calculated using the standard deviation. The LOD and LOQ were considered references of sensitivity and efficacy for the lateral flow immunoassay test strip/spectrum analyzer. These calculations were performed using the following formula:

LOD = *Blank* (mean) + 3 × *Blank* (standard deviation)

LOQ = *Blank* (mean) + 10 × *Blank* (standard deviation)

### 2.5. Neutralizing Antibody Inhibition Test of Wild-Type, Delta-Type, and Omicron-Type SARS-CoV-2

The ELISA-based neutralizing antibody inhibition test can be used to detect neutralizing antibodies specific to different types of SARS-CoV-2 in serum. Here, we used a commercially available test kit for wild-type (SARS-CoV-2 Surrogate Virus Neutralization Test Kit, GenScript, Piscataway, NJ, USA), Delta-type (AdipoGen Life Sciences, UK; Cat.: AG-48B-0007-KI01), and Omicron-type (Anti-SARS-CoV-2, B.1.1.529, Neutralizing Antibody Titer Serologic Assay Kit, AcroBiosystem, US; Cat.: RAS-N056) viruses and performed the protocol based on the manufacturer’s instructions. All of the above kits had high precision as Intra batch CV% (coefficient of variation) < 15% and Inter batch CV% < 15% with nearly 100% high specificity. The 96-well plates were precoated with recombinant protein ACE2 for wild-type SARS-CoV-2 Nab detection and spike proteins (receptor binding domain, specific to B.1.617.2 variant) for Delta-type and Omicron-type SARS-CoV-2 Nab detection, respectively. The samples and controls were diluted with wash buffer and added to the wells of the 96-well plates, followed by horseradish peroxidase (HRP)-conjugated receptor binding domain solution, and a 30-min incubation time at room temperature.

For wild-type Nab detection, only 15 min of incubation at room temperature was required, and supernatant removal with well washing was performed as per the provided protocol. For Delta-type and Omicron-type Nab detection, an hour of incubation at room temperature was required, and wells were subsequently washed five times, followed by the addition of ACE2-HRP solution, an additional hour of incubation, and a repeat of the washing steps. Finally, tetramethyl benzidine (TMB) substrate solution was added to all wells in the dark and the plates were incubated for 15 min before stop solution was added. The subsequent colorimetric change in each well was detected based on the O.D. value absorbance at 450 nm as read by a microtiter plate reader (Molecular Devices, San Jose, CA, USA).

The Nab inhibition percentage was then calculated with the O.D value results based on the following formula:

$$\mathrm{Inhibition}\;\%\;=\left(\;1-\frac{OD450\;value\;of\;sample}{average\;OD450\;value\;of\;negative\;control}\right)*100\%$$

### 2.6. Statistics

To verify the rapid test strip performance, the Spearman rank correlation coefficient was determined to analyze the correlation between the results from conventional ELISA and the results from the lateral flow immunoassay/spectrum analyzer for the maternal and fetal cord blood samples. An analysis was also performed to examine the differences in Nab values for wild-type or Delta-types of SARS-CoV-2 as determined by ELISA and by lateral flow immunoassay. The data were analyzed using GraphPad Prism, and the result of *p* < 0.05 was considered to be statistically significant.

## 3. Results

### 3.1. Participant Characteristics

A total of 20 pregnant women and 20 corresponding, pending newborns were enrolled in our study. All enrolled pregnant women were confirmed negative for COVID-19 infection during admission for delivery. The participants’ characteristics are provided in Table S1. All enrolled women had previously received at least one dose of a COVID-19 vaccine (Moderna mRNA-1273 vaccine). A total of 17 of the women enrolled had previously received two doses, and three of the women enrolled had received just one dose. The mean maternal age was 34 years (interquartile range, IQR 33–36), the mean gestational age of delivery was at 38.55 weeks (IQR 38–39), and the mean gestational age of the first and second doses of the vaccines was29.05 (IQR 27.75–30.0) and 33.71 (IQR 32.0–35.0) weeks, respectively. The mean BMI of mothers was 26.745 (range 24.05–28.85), and the mean baby body weight was 3155.75 g (range 2995.0–3376.25).

### 3.2. Neutralizing Antibodies in Maternal and Neonatal Serum

Table 1 indicates the Nab inhibition rates for wild-type and Delta-type SARS-CoV-2 from maternal peripheral blood and neonatal cord blood. Enrolled subjects that completed two doses of the COVID-19 vaccine before delivery had higher Nab inhibition rates for wild-type SARS-CoV-2 than those with only one dose of the vaccine for both maternal and neonatal serum taken during delivery (mothers: 97.45% vs. 40.32%, *p* = 0.062; neonates: 97.11% vs. 43.33%, *p* = 0.024). The Nab inhibition rates for Delta-type SARS-CoV-2 were quite low in maternal serum and neonatal cord blood for those with only one dose of the vaccine (mothers: 4.01%; neonates: 1.44%), and less than the cut-off value of 30% for the presence of Nabs [25]. However, the rates were elevated for samples from enrolled subjects receiving two vaccine doses (mothers: 77.82% vs. 4.01%, *p* < 0.001; neonates: 64.21% vs. 1.44%, *p* < 0.001). For wild-type SARS-CoV-2, no obvious difference in Nab inhibition rates for maternal serum or neonatal cord blood was detected during the interval between the second vaccine dose and delivery (0–2 weeks: approximately 95.99% and 95.78%; 4–8 weeks: approximately 97.48% and 97.56% for mothers and neonates, respectively), but for Delta-type, there was a trend toward higher Nab inhibition rates when the interval was longer (0–2 weeks: about 49.46% and 41.86%; 4–8 weeks: about 80.24% and 68.79% for mothers and neonates, respectively). Generally, Nab inhibition rates were significantly lower for Delta-type SARS-CoV-2 than wild-type in both maternal serum and neonatal cord blood (mothers: 64.88% vs. 97.34%, *p* < 0.001; neonates: 57.48% vs. 97.06%, *p* < 0.001). The results are shown in Table S2.

### 3.3. SRBD IgG Detection by Lateral Flow Immunoassay with Spectrum Analyzer

Figure 1A illustrates the mechanism of our rapid test strip based on a lateral flow immunoassay for the detection of SRBD IgG in human serum. Serum samples containing SRBD IgG produced two red lines (a test line and a control line) on the test strip. Additionally, higher SRBD IgG concentrations produced a more visible test line as indicated in Figure 1B. As shown in Figure 1C, and in a previous description, we used a spectrum analyzer equipped with a rapid test strip cassette for lateral flow immunoassays, and a quantitative measurement of color intensity by a spectrum reader was performed. As shown in Figure 1D, the reflectance spectra of SRBD IgG at approximately 540 nm was detected with significant differences between the test line and control line region. The standard curve of the spectrum analyzer with the lateral flow strip is provided in Figure 1E. The trend line equation was “y = 0.0002x + 0.9974, R² = 0.9771”. The LOD and LOQ were 105.89 ng/mL and 248.41 ng/mL, respectively. The calculation followed our previous publication [24], and the details are described in the supporting information.

**Figure 2:**
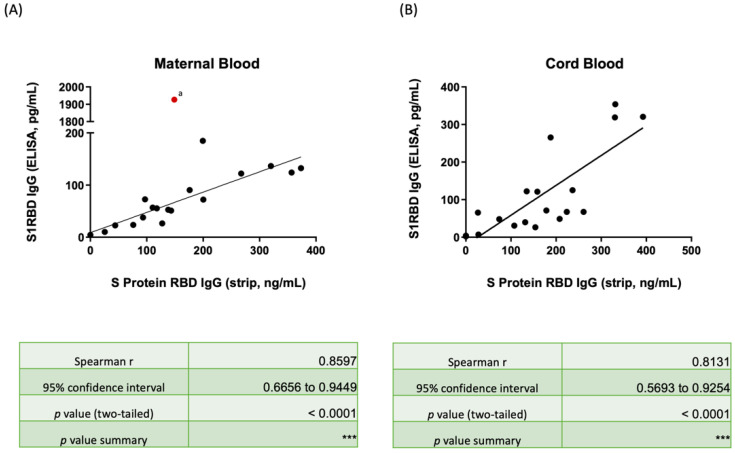
(**A**) Scatter plot showing the correlation between SRBD IgG by the lateral flow immunoassay/spectrum analyzer and ELISA for maternal serum. (**B**) Scatter plot showing the correlation between SRBD IgG by the lateral flow immunoassay/spectrum analyzer and ELISA in neonatal cord blood. The red spot (a) in Table S3 was case 17 and was censored in the correlation line due to it being an outlier. SRBD, spike protein receptor binding domain; ELISA, enzyme-linked immunosorbent assay. *** *p* < 0.001.

Figure 2 shows the correlation between the S1RBD IgG concentration in maternal and neonatal serum as determined by ELISA, and the SRBD IgG results as determined by the spectrum reflectance using our lateral flow immunoassay/spectrum analyzer. Among those subjects that received two vaccine doses, the median SRBD level was 143.26 ng/mL (IQR 103.84–233.87) and 178.3 ng/mL (IQR 119.44–248.82) for mothers and neonates, respectively. Furthermore, a significantly lower SRBD IgG level was detected in the subjects that had only received one dose of the vaccine (mothers: 8.58 ng/mL, *p* = 0.010; neonates: 9.28 ng/mL, *p* = 0.007). Similar results were found via ELISA for both mothers (2 doses: 72.11 pg/mL, 1 dose: 4.45 pg/mL), and neonates (2 doses: 67.65 pg/mL; 1 dose: 4.29 pg/mL). The correlation between the two detection methods was highly relevant with significance in both maternal serum (Rho = 0.8597, *p* value < 0.0001) and neonatal cord blood (Rho = 0.8131, *p* value < 0.0001). All results are provided in Table S3.

### 3.4. Nab Inhibition Rates of Wild-, Delta-, and Omicron-Type SARS-CoV-2 with SRBD IgG Level

**Figure 3:**
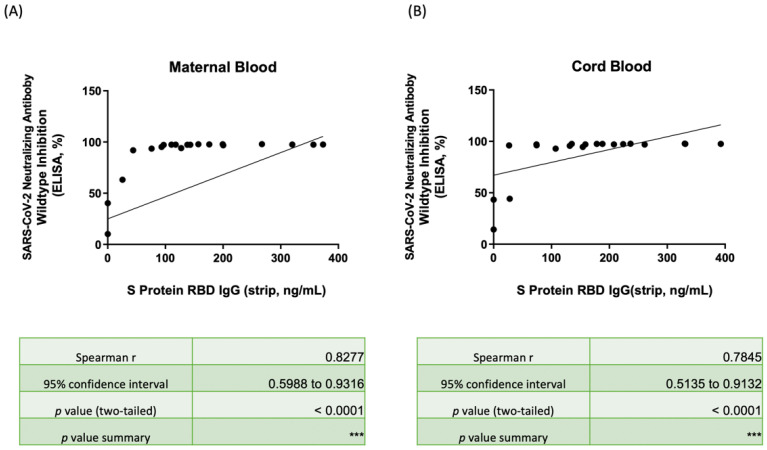
(**A**) Scatter plot showing the correlation between SRBD IgG by the lateral flow immunoassay/spectrum analyzer and the neutralizing antibody inhibition rates against wild-type SARS-CoV-2 by ELISA in maternal serum. (**B**) Scatter plot showing the correlation between SRBD IgG by the lateral flow immunoassay/spectrum analyzer and the neutralizing antibody inhibition rates against wild-type SARS-CoV-2 by ELISA in neonatal cord blood. SRBD, spike protein receptor binding domain; ELISA, enzyme-linked immunosorbent assay. *** *p* < 0.001.

Figure 3 illustrates the correlation between the Nab inhibition percentage of maternal serum (Figure 3A) and neonatal cord blood (Figure 3B) for wild-type SARS-CoV-2 and the SRBD IgG result based on the spectrum reflectance using our lateral flow immunoassay/spectrum analyzer. The complete results are listed in Table S4. Only the three participants with only one dose of the vaccine had Nab inhibition rates less than 90% in both maternal/neonatal blood (mothers: 10.24%, 40.32%, and 63.15%; neonates: 14.28%, 43.33%, and 44.14%), and notably lower SRBD IgG concentration was also detected (mothers: 25.73, 0, and 0 ng/mL by strip, 10.19, 4.07, and 4.45 pg/mL by ELISA; neonates: 27.84, 0, and 0 ng/mL by strip, 7.22, 4.29, and 2.25 pg/mL by ELISA). The rho was 0.8277 and 0.7845 in maternal serum and neonatal cord blood with significance (both *p* value < 0.001), respectively, which both showed positive relevancy.

**Figure 4:**
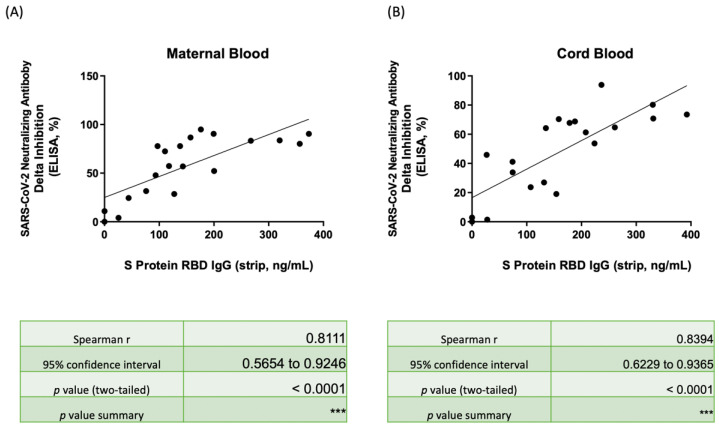
(**A**) Scatter plot showing the correlation between SRBD IgG by the lateral flow immunoassay/spectrum analyzer and the neutralizing antibody inhibition rates against Delta-type SARS-CoV-2 by ELISA in maternal serum. (**B**) Scatter plot showing the correlation between SRBD IgG by the lateral flow immunoassay/spectrum analyzer and the neutralizing antibody inhibition rates against Delta-type SARS-CoV-2 by ELISA in neonatal cord blood. SRBD, spike protein receptor binding domain; ELISA, enzyme-linked immunosorbent assay. *** *p* < 0.001.

**Figure A1:**
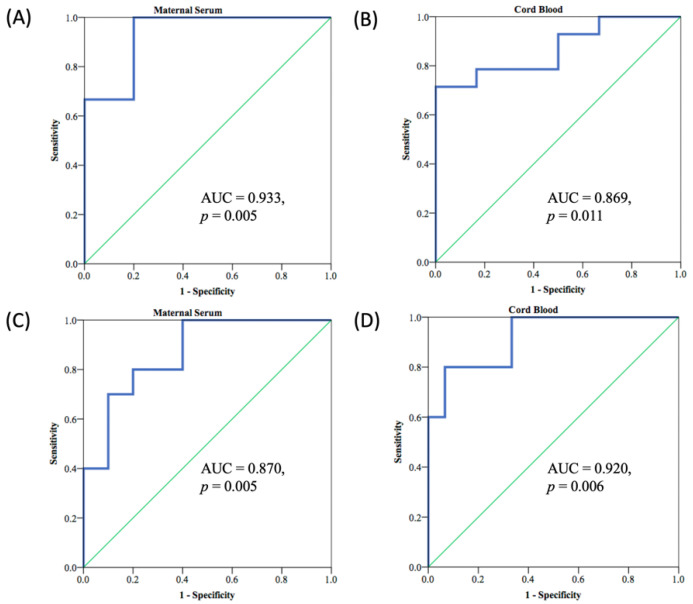
(**A**) ROC curve of S1RBD in maternal serum by the immunoassay/spectrum analyzer for Nab inhibition rate ≧ 30% for Delta-type SARS-CoV-2. (**B**) ROC curve of S1RBD in neonatal cord blood by the immunoassay/spectrum analyzer for Nab inhibition rate ≧ 30% for Delta-type SARS-CoV-2. (**C**) ROC curve of S1RBD in maternal serum by the immunoassay/spectrum analyzer for Nab inhibition rate ≧ 70% for Delta-type SARS-CoV-2. (**D**) ROC curve of S1RBD in neonatal cord blood by the immunoassay/spectrum analyzer for Nab inhibition rate ≧ 70% for Delta-type SARS-CoV-2. ROC curve, receiver operating characteristic curve; SRBD, spike protein receptor binding domain; AUC, area under curve.

**Figure A2:**
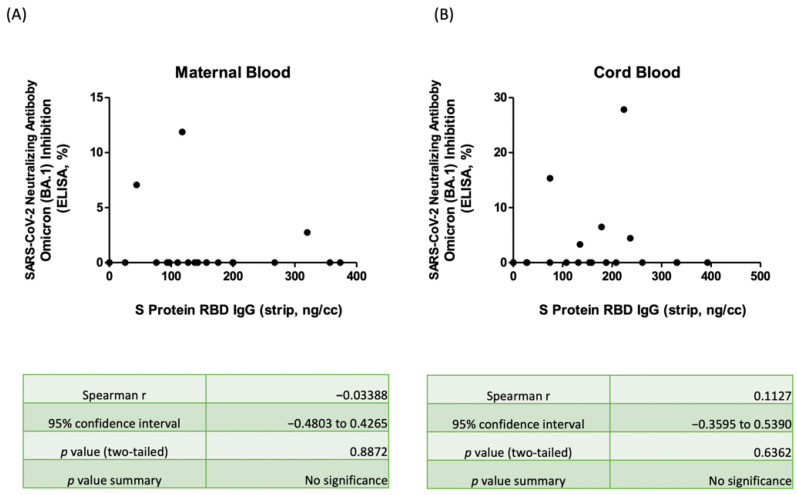
(**A**) Scatter plot showing the correlation between SRBD IgG by the lateral flow immunoassay/spectrum analyzer and the neutralizing antibody inhibition rates against Omicron-type SARS-CoV-2 by ELISA in maternal serum. (**B**) Scatter plot showing the correlation between SRBD IgG by the lateral flow immunoassay/spectrum analyzer and the neutralizing antibody inhibition rates against Omicron-type SARS-CoV-2 by ELISA in neonatal cord blood. SRBD, spike protein receptor binding domain; ELISA, enzyme-linked immunosorbent assay.

Figure 4 illustrates the correlation between the Nab inhibition percentage for Delta-type SARS-CoV-2 and the SRBD IgG result as determined by the lateral flow immunoassay strip for maternal serum (Figure 4A) and neonatal cord blood (Figure 4B). The data are provided in Table S5. The Nab inhibition rates were diverse and had positive relevancy for SRBD IgG concentration. The median Nab inhibition rates for Delta-type in maternal and neonatal blood were 64.88% and 57.48%; this was lower than the inhibition rates found for wild-type, which were 97.34% (*p* < 0.001) and 97.06% (*p* < 0.001), respectively. Samples from the three subjects that had only received one dose of the vaccine demonstrated significantly poorer Nab inhibition rates than the subjects that received two full doses of the vaccine (mothers: 77.82% vs. 4.01%, *p* < 0.001; neonates: 64.21% vs. 1.44%, *p* < 0.001). Among the subjects that received two vaccine doses, 88.2% and 82.3% of mothers and neonates, respectively, had Nab inhibition rates over 30%. As shown in Table S6, the cut-off value of SRBD IgG for Nab inhibition rates to Delta-type over 30% (as measured by the lateral flow immunoassay) was 60.15 ng/mL (area under curve, AUC: 0.933, *p* = 0.005) (Figure A1A) and 156.31 ng/mL (AUC: 0.869, *p* = 0.011) (Figure A1B) for mothers and babies, respectively. Additionally, the cut-off value of SRBD IgG for Nab inhibition rates to Delta-type over 70% can be detected as 150.21 ng/mL (area under curve, AUC: 0.870, *p* = 0.005) (Figure A1C) and 230.20 ng/mL (area under curve, AUC: 0.920, *p* = 0.006) (Figure A1D) for mothers and babies, respectively. The rho results were 0.8111 and 0.8394 in maternal and neonatal blood, respectively, and both were with significance (*p* < 0.0001). Table S7 demonstrated the Nab inhibition rates for Omicron-type, and both the median values in maternal serum and neonatal cord blood were only 0, which were much lower than in wild/delta-type SARS-CoV-2. The lower correlation between Nab inhibition rates of maternal serum (Figure A2A) and neonatal cord blood (Figure A2B) for Omicron-type SARS-CoV-2 and the SRBD IgG detected by the lateral flow test also found that the rho was −0.03388 and 0.1127 in maternal serum and neonatal cord blood (both *p* value no significance), respectively.

### 3.5. Correlation of Nab Concentration and SRBD IgG Level

**Figure 5:**
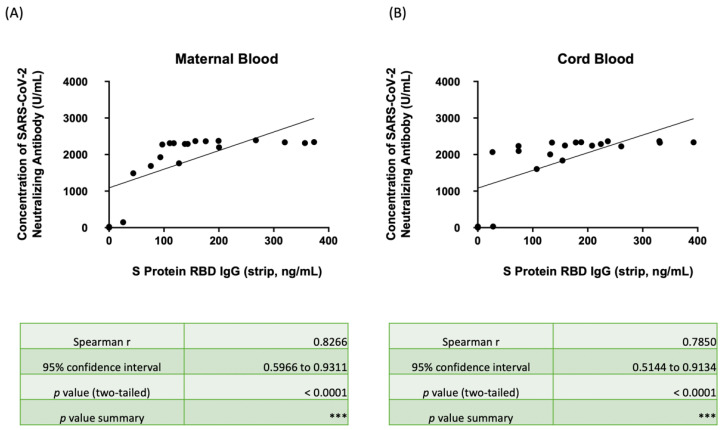
(**A**) Scatter plot showing the correlation between SRBD IgG by the lateral flow immunoassay/spectrum analyzer and the neutralizing antibody concentration against wild-type SARS-CoV-2 by ELISA in maternal serum. (**B**) Scatter plot showing the correlation between SRBD IgG by the lateral flow immunoassay/spectrum analyzer and the neutralizing antibody concentration against wild-type SARS-CoV-2 by ELISA in neonatal cord blood. SRBD, spike protein receptor binding domain; ELISA, enzyme-linked immunosorbent assay. *** *p* < 0.001.

Figure 5 illustrates the correlation between the Nab concentration for wild-type SARS-CoV-2 by ELISA and the S1RBD IgG concentration by the lateral flow immunoassay strip in maternal serum (Figure 5A) and neonatal cord blood (Figure 5B). The complete data are listed in Table S8. The median Nab concentration was 2292.03 U/mL (IQR 1706.50–2338.98) and 2239.63 U/mL (IQR 1879.58–2330.41) in maternal and neonatal blood, respectively. Among the participants, 65% of the mothers and 80% of the babies had serum Nab concentrations over 2000 U/mL. Furthermore, the three single-vaccine injected subjects also had much lower serum Nab concentrations (maternal: 2.21, 24.30, 149.98 U/mL; neonatal: 3.05, 30.89, 32.97 U/mL, respectively). In total, 92.4% of those mothers with SRBD IgG concentrations over 100 ng/mL, as determined by the lateral flow immunoassay, had serum Nab concentrations over 2000 U/mL, but one case with a maternal serum SRBD IgG of 127.55 ng/mL only had a serum Nab concentration of 1761.85 U/mL. In total, 86.7% of those babies with an SRBD IgG concentration over 100 ng/mL had serum Nab concentrations over 2000 U/mL, but two babies with a SRBD IgG concentration of 107.25 and 154.12 ng/mL had only 1606.50 and 1838.23 U/mL of serum Nab concentrations, respectively. The rho of correlations was 0.8266 and 0.7850 in maternal and serum concentrations, respectively, and both showed positive relevancy with significance (*p* < 0.001).

## 4. Discussion

The COVID-19 pandemic has produced a massive influence and impact on the economic and daily life of people, societies, and countries worldwide. As of January 2022, over 300 million people have become infected and five million people have succumbed to the disease [26]. The development of vaccines and wide promotion of vaccination can effectively produce and upregulate virus antibody in the human body to generate protection against SARS-CoV-2, which is especially impactful for physically at-risk individuals and those with vulnerable cohorts, such as healthcare workers and pregnant women [27,28,29]. However, the potentially endless emergence of variant types of SARS-CoV-2, such as Alpha (B.1.1.7), Beta (B.1.351), Gamma (P.1), Delta (B.1.617.2), and Omicron (B.1.1.529) variants, reduces vaccine efficacy, and numerous breakthrough SARS-CoV-2 infections after vaccination have been reported. Vaccine protection evaluation can be determined by analyzing neutralizing antibodies (Nabs) concentration or inhibition rates to virus protein [30]. The antibody for the spike protein receptor binding domain (SRBD IgG) to SARS-CoV-2 is a well-established marker that can be used to assess Nab detection [16,31]. From the literature, the antibody tests (IgG/IgM) showed low sensitivity (less than 30.1%) in the first week of the onset of symptoms, but increased up to 72.2%, 91.4%, and 96% during the second week, third week, and fourth-to-fifth weeks, respectively [21]. Additionally, the overall sensitivity was around 70 to 80% by chemiluminescence immunoassay and ELISA with IgG or IgM. The lateral flow immunoassay showed sensitivity as 78%, 47%, and 82% with IgG, IgM, and IgM/IgG [32], respectively. In our previous research, the use of a lateral flow immunoassay in combination with a spectrum analyzer has been shown to effectively detect IgG in serum [17].

The examined cohorts were different between the present study and our previous study [33]. In the present study, we have focused on vulnerable cohorts, i.e., pregnant women and their neonates, which have not been extensively discussed in academic articles. In addition, in our previous study, we derived a mathematical formula for describing the color intensity (recorded via our spectrum-based reader) between the control line region and test line region of the lateral flow immunoassay. The reflectance spectra (of our spectrum-based reader) were used to acquire a value for constructing a neutralizing antibody concentration, which was equivalent to the percentage of inhibition (not the neutralizing antibody amount) [33]. Our current study has attempted to use a lateral flow immunoassay (coupled with our spectrum-based reader) to quantify the amount of neutralizing antibodies, which is the main difference from our previous study. We have further taken this technology advantage to conclude that an additional vaccine booster may be considered for those mothers with the specific neutralizing antibody level.

As with previous research [17], our study demonstrated the suitability for a lateral flow immunoassay to be used in combination with a spectrum analyzer to detect the SRBD IgG level in pregnant women’s serum and neonatal cord blood as noted in Figure 2 (mothers: Rho = 0.8602, *p* < 0.0001; neonates: Rho = 0.8135, *p* < 0.0001). Beharier et al., reported that SRBD IgG transfer across the placental barrier can be triggered by the BNT162b2 mRNA vaccine within 15 days after administration of the first vaccine dose [34]. Matsui et al., reported that neonates can also obtain SARS-CoV-2 Nabs from their vaccinated mothers, and the transfer ratio (cord Nab level/maternal Nab level) was greatest when vaccination was completed in the second trimester (1.7-fold higher, *p* < 0.0001) [35]. Our previous study also showed considerable levels of Nab inhibition rates can be detected in both maternal serum and neonatal cord blood from fully vaccinated pregnant women, and both levels were higher than in those who had only received a single vaccine dose (maternal: 97.46% vs. 4.01%; neonatal: 97.37% vs. 1.44%) [14]. A high correlation in Nab inhibition rates with positive relevancy between maternal and cord blood has been detected (Rho = 0.7669, *p* < 0.0001) [14]. Here, we found that median Nab inhibition rates for wild-type SARS-CoV-2 were 97.34% (IQR 93.65–97.60) and 97.06% (IQR 94.85–97.55) in maternal serum and neonatal cord blood, respectively, which were both higher than the results for Delta-type (mothers: 64.88%, *p* < 0.001; neonatal: 57.48%, *p* < 0.001) and Omicron-type SARS-CoV-2 (both 0 in mothers and neonates, *p* < 0.001) with significance. The cord-to-maternal ratio was approximately 1.30 (IQR 0.78–2.23) and 0.71 (IQR 0.51–1.06) for the subjects that received two vaccine doses and those that received one dose, respectively. These data provide additional support for providing two vaccine doses for pregnant women and note that the Nab protection provided can be transferred to newborns. We found that this Nab protection was greatest when the interval between receiving the second vaccine dose and delivery was four-to eight-weeks, which is a finding supported by other articles as well [36,37,38].

As shown in Figure 3, the subjects enrolled in our study received the Moderna (mRNA-1273) vaccine and demonstrated excellent immunity to wild-type SARS-CoV-2 with median Nab inhibition rates of 97.34% and 97.06% for mothers and babies, respectively. Those receiving only one dose of the vaccine had relatively lower Nab protection, as low as 40.32% and 43.33% for mothers and neonates, respectively. However, all subjects that received two vaccine doses demonstrated Nab inhibition rates of at least 90% and Nab concentrations of at least 1400 U/mL in both mothers and neonates. Additionally, the values were similar in regard to protection for Delta-type SARS-CoV-2. Those subjects that received two vaccine doses demonstrated Nab protection that was 77.82% and 64.21% higher than those that received only one vaccine dose (4.01% and 1.44% for mothers and neonates, respectively). As shown in Figure A1, the SRBD IgG cut-off value for Nab inhibition rates of at least 30% for Delta variants was 60.15 and 156.31 ng/mL in mothers and babies, respectively. This means that mothers or neonates may need additional vaccine doses. These results may help guide vaccine strategy for possible booster vaccinations. For the neonates with lower SRBD IgG, close protection with special care may be needed since there is no available vaccine for this cohort. The cut-off value of SRBD IgG for Nab inhibition rates of at least 70% for Delta variants was 150.21 and 230.20 ng/mL for mothers and babies, respectively. The Nab inhibition rates which ranged within 30 to 70% can indicate moderate vaccine protection. Thus, the results of SRBD IgG from a lateral flow strip with a spectrum analyzer may give us an immediate message that the vaccine protection is sufficient for some variants of SARS-CoV-2. Examining the SRBD IgG level with a rapid lateral flow immunoassay test may provide a much-needed and integral tool for point-of-care testing and vaccine guidance.

From the previously published literature, the reduction and loss of vaccine protection may be related to weakened self-immunity or health status for antibody generation, different variants of SARS-CoV-2 that breach established immunity, diminished vaccine efficacy over time, and unimplemented physical barriers against virus infection. Additionally, the literature has shown that protection provided by a COVID-19 vaccine may be weakened six months after vaccination, and a breakthrough infection with a drop in antibody amount may be observed. COVID-19 booster vaccines have been found to enhance protection against SARS-CoV-2 and reduce the likelihood of subsequent severe illness [39,40,41,42]. Because of the limited sample size in this study, no significant differences in the SRBD IgG level or Nab inhibition rates could be detected among our cohorts for different parameters or variates of clinical data, including age of delivery, gestational age of delivery, BMI status, neonatal body weight, or neonatal gender. Those subjects that received two vaccine doses demonstrated greater protection than those that received only one dose, and those with an interval between the second dose and delivery of at least two weeks demonstrated better immunity than those with an interval of less than two weeks. Considering the variants of SARS-CoV2 and breakthrough infections following complete vaccination [43,44], it is important to evaluate the SRBD IgG level as a measure of immunity against COVID-19, and the lateral flow immunoassay strip test is a preferred tool for rapid, simplified testing that can guide decisions regarding booster vaccination needs. In our current study, the working duration for our lateral flow immunoassay was about 7–10 min, and the analysis duration for our spectrum-based optical analyzer was about 3–5 min.

There were some limitations in our study. Because only 20 participants and 40 samples were used, the range and breadth of results in terms of clinical differences were limited. Additionally, because vaccine coverage is expanding in the population at large, it was difficult to find high numbers of pregnant women that were vaccinated after the onset of pregnancy, and the quality of the samples and lateral flow strip may affect both the examination and the calculation of the LOD. Other limitations included the fact that we only enrolled subjects that had received a particular vaccine, Moderna (mRNA-1273), and we only tested for protection against wild-type and Delta-type SARS-CoV-2. Additional variants may be studied in the future.

## 5. Conclusions

In this study, we found that COVID-19 vaccine protection in pregnant women and neonates was better for wild-type virus compared to Delta-type and Omicron-type SARS-CoV-2 virus. Additionally, enrolled subjects that received two vaccine doses demonstrated better protection than those that received only one dose. We further found that a lateral flow immunoassay/spectrum analyzer approach could be used to detect the S1RBD IgG level cut-off value in maternal and neonatal serum, which was 60.15 and 156.31 ng/mL for Nab inhibition rates of at least 30% against Delta-type SARS-CoV-2. For those mothers with an S1RBD IgG level less than 60.15 ng/mL, an additional vaccine booster may be considered to augment low immunity/protection. For those neonates with an S1RBD IgG level less than 156.31 ng/mL, close protection with special care may be needed since there is no available vaccine for them. More cases may be needed in a future study for further clinical validation.

## Figures and Tables

**Table 1:** Neutralizing antibodies against wild-type and Delta-type SARS-CoV-2 in maternal serum and neonatal cord blood *.

Characteristics	One-Dose Group (Median, IQR)	Two-Dose Group (Median, IQR) (Interval of Second Administration to Delivery)	0–2 Weeks	2–4 Weeks	4–8 Weeks
Maternal SRBD IgG by Strip and spectrum analyzer	0 ng/mL ** (0–12.87)	143.27 ng/mL *** (110.61–200.35)	146.91 ng/mL ^1^ (120.19–173.63)	127.99 ng/mL ^2^ (102.28–184.02)	157.14 ng/mL ^3^ (127.55–267.39)
Cord blood SRBD IgG by Strip and spectrum analyzer	0 ng/mL ** (0–13.92)	178.40 ng/mL *** (131.62–236.61)	207.58 ng/mL ^1^ (180.85–234.31)	146.75 ng/mL ^2^ (114.19–195.52)	188.03 ng/mL ^3^ (131.62–330.32)
Maternal neutralizing antibody (Wild-type)	40.32% ** (25.28–51.74)	97.45% *** (96.82–97.61)	95.99% ^1^ (95.58–96.41)	97.39% ^2^ (97.26–97.54)	97.48% ^3^ (97.35–97.74)
Cord blood neutralizing antibody (Wild-type)	43.33% ** (28.80–43.74)	97.11% *** (96.24–97.56)	95.78% ^1^ (95.18–96.38)	97.10% ^2^ (96.31–97.28)	97.56% ^3^ (97.03–97.59)
Cord to maternal ratio (Wild-type)	1.07 ** (0.89–1.23)	0.999 *** (0.996–1.00)	0.99 ^1^ (0.99–1.01)	1.00 ^2^ (0.99–1.00)	1.00 ^3^ (1.00–1.00)
Maternal neutralizing antibody (Delta-type)	4.01% ** (0.87–7.51)	77.82% **** (52.13–83.63)	49.96% ^1^ (48.87–51.06)	79.27% ^2^ (62.42–82.90)	80.24% ^3^ (59.98–86.71)
Cord blood neutralizing antibody (Delta-type)	1.44% ** (2.16)	64.21% **** (41.14–70.38)	41.86% ^1^ (30.46–53.26)	57.48% ^2^ (47.82–63.47)	68.79% ^3^ (41.14–73.56)
Cord to maternal ratio (Delta-type)	0.92 ** (0.86–1.40)	0.87 **** (0.77–0.94)	0.86 ^1^ (0.61–1.11)	0.78 ^2^ (0.75–0.85)	0.92 ^3^ (0.85–0.94)

SRBD, spike protein receptor binding domain; IQR, interquartile range. * Median ratio of neutralizing antibodies against Omicron-type SARS-CoV-2 was 0 in mothers and neonates, so no further comparison was done; ** Case number of one-dose group = 3; *** Case number of two-dose group = 17; **** Case number of Delta variant group = 20; ^1^ Case number = 2; ^2^ Case number = 6; ^3^ Case number = 9.

## Data Availability

Not applicable.

## Supplementary Materials

The following supporting information can be downloaded at: https://www.mdpi.com/article/10.3390/bios12100891/s1, Supportive information. The calculation of LOD and LOQ; Table S1. Characteristics of Maternal and neonatal status; Table S2. Median neutralizing antibody inhibition rates to wild or Delta-type SARS-CoV-2 in maternal serum and neonatal cord blood; Table S3. SRBD IgG level by lateral flow immunoassay and ELISA in maternal serum and neonatal cord blood; Table S4. SRBD IgG level by lateral flow immunoassay and neutralizing antibody inhibition rates against wild-type SARS-CoV-2 in maternal serum and neonatal cord blood; Table S5. SRBD IgG level by lateral flow immunoassay and neutralizing antibody inhibition rates against Delta-type SARS-CoV-2 in maternal serum and neonatal cord blood; Table S6. The ROC curve result and cut-off value of SRBD IgG for neutralizing antibody inhibition rates over 30% by lateral flow immunoassay/spectrum analyzer in maternal serum and neonatal cord blood; Table S7. SRBD IgG level by lateral flow immunoassay and neutralizing antibody inhibition rates against Omicron-type SARS-CoV-2 in maternal serum and neonatal cord blood; Table S8. SRBD IgG level by lateral flow immunoassay and neutralizing antibody concentration against wild-type SARS-CoV-2 by ELISA in maternal serum and neonatal cord blood.
