# Supplementary material for: Detection of SARS-CoV-2 Neutralizing Antibodies in Vaccinated Pregnant Women and Neonates by Using a Lateral Flow Immunoassay Coupled with a Spectrum-Based Reader
Source: Biosensors (Basel). 2022 Oct 18;12(10):891. doi: 10.3390/bios12100891 (PMC9599695; doi:10.3390/bios12100891)
Supplement: Supplementary file 1 [file biosensors-12-00891-s001.zip › biosensors-1864236-supplementary.pdf]

The calculation of LOD and LOQ

The trend line equation of figure 1E is y=0.0002x+0.9974 with R2=0.9771. We have revised it in our revised manuscript. To obtain the LOD and LOQ values, we need to have both mean and standard deviation of blank samples based on the equations that we mentioned on lines 208-209. The trend line of Figure 1E was calculated from the data as following:

ng/ml	1	2	3	4	5	6	7
0	0.99846	1.006239	1.008437	1.002306	1.007	1.012093	1.007205
5	1.003273	1.008345	1.01483	1.010921	1.007818	1.004264	1.002545
10	0.99953	1.000562	1.001061	1.002078	1.005969	0.999878	1.011188
20	1.002988	0.998385	1.008905	1.002081	1.005295	1.007142	0.994364
30	1.007405	0.997749	1.003941	1.000851	1.002942	1.012689	1.000968
50	1.004689	1.001583	1.003845	1.005316	1.011383	1.004449	1.000543
75	1.01748	1.007918	1.013957	1.011785	1.009691	1.012268	1.019396
100	1.015706	1.014117	1.016057	1.021859	1.017264	1.014285	1.019916
200	1.036708	1.037997	1.037958	1.025697	1.050204	1.030301	1.060621
300	1.06447	1.065788	1.051624	1.064209	1.06898	1.068272	1.062782
400	1.090305	1.078092	1.046831	1.044127	1.114717	1.019364	1.266916

The concentration of blank samples was 0 ng/ml. The mean of blank samples was 1.005963, calculated by the equation "sum / numbers". The standard deviation was 0.004072, calculated based on the equation as following:

$$s = \sqrt{\frac{\sum(x - \bar{x})^2}{n - 1}}$$

Thus, we could have both LOD and LOQ as 1.018179 and 1.046683 based on the below equations:

$$LOD = Blank\ (\text{mean}) + 3 \times Blank\ (\text{standard deviation})$$

$$LOQ = Blank\ (\text{mean}) + 10 \times Blank\ (\text{standard deviation})$$

We then have real LOD and LOQ as 105.893 and 248.41 ng/ml by using the trend line equation as "y=0.0002x+0.9974". The reference of calculation can be seen on our previous publication (reference 1).

Reference: Lin, S. W., Shen, C. F., Liu, C. C., & Cheng, C. M. (2021). A Paper-Based IL-6 Test Strip Coupled With a Spectrum-Based Optical Reader for Differentiating Influenza Severity in Children. *Frontiers in bioengineering and biotechnology*, *9*, 752681.

Table S1. Characteristics of Maternal and neonatal status

Variable	Included in the Analysis
Age of mothers (years)[a]	34.05* (±4.06**), IQR 36-33
Parity[a] ≧1	17 (85%***), IQR 1-0
BMI[a]	26.745* (±4.13**), IQR 28.85-24.05
Weeks of gestation at the first dose of COVID-19 vaccination (weeks)[a]	29.05* (±2.46**), IQR 30-27.75
Weeks of gestation at the second dose of COVID-19 vaccination (weeks)[b]	33.71* (±2.28**), IQR 35-32
Interval between the second dose of COVID-19 vaccination and the collection of blood samples (day of delivery) (weeks)[b]	4.76* (±1.99**), IQR 6-4
Interval between the first dose of COVID-19 vaccination and the collection of blood samples (day of delivery) (weeks)[a]	9.35* (±2.28**), IQR 11-8
Weeks of gestation at delivery (weeks)[a]	38.55* (±1.10**), IQR 39-38
Gender of newborn[a]	
Male	9 (45%***)
Female	11 (55%***)
Weight of newborn (g)[a]	3155.75* (±352.16**), IQR 3376.25-2995

IQR, *interquartile range; BMI, body mass index; COVID-19, Coronavirus* disease 2019. * Mean; ** Standard Deviation (±SD); *** Percentage of all surveyed patients; [a] Case number = 20; [b] Case number = 17.

Table S2. Median neutralizing antibody inhibition rates to wild or Delta-type SARS-CoV-2 in maternal serum and neonatal cord blood.

	Mother (%)	Baby (%)
Wild-type	97.34 (IQR 93.65 – 97.60)	97.06 (IQR 94.85 – 97.55)
Delta-type	64.88 (IQR 29.31 – 83.56)	57.48 (IQR 24.49 – 69.98)
Omicron-type	0	0 (IQR 0 – 2.48)
P value	< 0.001	< 0.001

IQR, interquartile range.

Table S3. SRBD IgG level by lateral flow immunoassay and ELISA in maternal serum and neonatal cord blood

Case	Vaccine Dose	SRBD IgG by LFI with SR (mothers) (ng/ml)	S1RBD IgG by ELISA (mothers) (pg/ml)	SRBD IgG by LFI with SR (neonates) (ng/ml)	S1RBD IgG by ELISA (neonates) (pg/ml)
1	2	200.35	72.11	154.12	26.49
2	2	356.83	123.94	392.56	320.51
3	2	267.39	122.13	331.32	353.72
4	2	76.17	23.59	107.25	30.87
5	2	117.91	55.30	223.79	67.57
6	2	97.07	72.55	207.86	48.73

7	2	138.09	52.71	158.50	121.09
8	2	143.27	50.94	74.03	48.08
9	1	0.00	4.45	0.00	2.25
10	2	176.04	90.50	236.61	125.08
11	2	127.55	26.46	131.62	39.95
12	2	110.61	56.70	178.40	71.08
13	2	44.14	22.80	74.42	49.14
14	2	199.34	184.70	26.82	65.64
15	2	320.01	136.56	134.99	122.22
16	2	373.35	132.37	330.32	318.82
17	2	157.14	1912.73	188.03	265.61
18	1	0.00	4.07	0.00	4.29
19	1	25.73	10.19	27.84	7.22
20	2	93.46	37.77	261.04	67.65

SRBD, spike protein receptor binding domain; LFI, lateral flow immunoassay; SR, spectrum reader; ELISA, enzyme-linked immunosorbent assay.

Table S4. SRBD IgG level by lateral flow immunoassay and neutralizing antibody inhibition rates against wild-type SARS-CoV-2 in maternal serum and neonatal cord blood.

Case	Vaccine Dose	SRBD IgG by LFI with SR (mothers) (ng/ml)	Nab inhibition rates (wild-type) (mothers) (%)	SRBD IgG by LFI with SR (neonates) (ng/ml)	Nab inhibition rates (wild-type) (neonates) (%)
1	2	200.35	96.82	154.12	94.58
2	2	356.83	97.48	392.56	97.58
3	2	267.39	97.88	331.32	97.53
4	2	76.17	93.51	107.25	92.89
5	2	117.91	97.45	223.79	97.33
6	2	97.07	97.24	207.86	97.08
7	2	138.09	97.34	158.50	97.11
8	2	143.27	97.35	74.03	97.04
9	1	0.00	10.24	0.00	14.28
10	2	176.04	97.74	236.61	97.75
11	2	127.55	94.05	131.62	95.66
12	2	110.61	97.45	178.40	97.56
13	2	44.14	91.94	74.42	96.25
14	2	199.34	97.79	26.82	96.06
15	2	320.01	97.57	134.99	97.54
16	2	373.35	97.61	330.32	97.76
17	2	157.14	97.75	188.03	97.59
18	1	0.00	40.32	0.00	43.33
19	1	25.73	63.15	27.84	44.15
20	2	93.46	95.17	261.04	96.98

SRBD, spike protein receptor binding domain; LFI, lateral flow immunoassay; SR, spectrum reader; Nab, neutralizing antibody.

Table S5. SRBD IgG level by lateral flow immunoassay and neutralizing antibody inhibition rates against Delta-type SARS-CoV-2 in maternal serum and neonatal cord blood.

Case	Vaccine Dose	SRBD IgG by LFI with SR (mothers) (ng/ml)	Nab inhibition rates (Delta-type) (mothers) (%)	SRBD IgG by LFI with SR (neonates) (ng/ml)	Nab inhibition rates (Delta-type) (neonates) (%)
1	2	200.35	52.15	154.12	19.05
2	2	356.83	80.25	392.56	73.56
3	2	267.39	83.35	331.32	70.82
4	2	76.17	31.61	107.25	23.68
5	2	117.91	57.29	223.79	53.69
6	2	97.07	77.82	207.86	61.26
7	2	138.09	77.82	158.50	70.38
8	2	143.27	56.98	74.03	41.14
9	1	0.00	-2.26	0.00	-4.26
10	2	176.04	94.95	236.61	93.86
11	2	127.55	28.54	131.62	26.93
12	2	110.61	72.48	178.40	67.83
13	2	44.14	24.39	74.42	33.91
14	2	199.34	90.47	26.82	45.86
15	2	320.01	83.63	134.99	64.21
16	2	373.35	90.41	330.32	80.21
17	2	157.14	86.71	188.03	68.79
18	1	0.00	11.00	0.00	2.88
19	1	25.73	4.01	27.84	1.44
20	2	93.46	47.77	261.04	64.67

SRBD, spike protein receptor binding domain; LFI, lateral flow immunoassay; SR, spectrum reader; Nab, neutralizing antibody.

Table S6. The ROC curve result and cut-off value of SRBD IgG for neutralizing antibody inhibition rates over 30% by lateral flow immunoassay/spectrum analyzer in maternal serum and neonatal cord blood

	Mother	Baby
For 30% inhibition rates		
Cut-off value	60.15	156.31
ROC curve	0.933	0.869
P value	0.005	0.011
For 70% inhibition rates		
Cut-off value	150.21	230.20
ROC curve	0.870	0.920
P value	0.005	0.006

ROC, receiver operating characteristic curve; SRBD, spike protein receptor binding domain.

Table S7. SRBD IgG level by lateral flow immunoassay and neutralizing antibody inhibition rates against Omicron-type SARS-CoV-2 in maternal serum and neonatal cord blood.

Case	Vaccine Dose	SRBD IgG by LFI with SR (mothers) (ng/ml)	Nab inhibition rates (Delta-type) (mothers) (%)	SRBD IgG by LFI with SR (neonates) (ng/ml)	Nab inhibition rates (Delta-type) (neonates) (%)
1	2	200.35	0	154.12	0
2	2	356.83	0	392.56	0
3	2	267.39	0	331.32	0
4	2	76.17	0	107.25	0
5	2	117.91	11.88	223.79	27.83
6	2	97.07	0	207.86	0
7	2	138.09	0	158.50	0
8	2	143.27	0	74.03	0
9	1	0.00	0	0.00	0
10	2	176.04	0	236.61	4.43
11	2	127.55	0	131.62	0
12	2	110.61	0	178.40	6.48
13	2	44.14	7.07	74.42	15.35
14	2	199.34	0	26.82	0
15	2	320.01	2.74	134.99	3.30
16	2	373.35	0	330.32	0
17	2	157.14	0	188.03	0
18	1	0.00	0	0.00	0
19	1	25.73	0	27.84	0
20	2	93.46	0	261.04	0

SRBD, spike protein receptor binding domain; LFI, lateral flow immunoassay; SR, spectrum reader; Nab, neutralizing antibody.

Table S8. SRBD IgG level by lateral flow immunoassay and neutralizing antibody concentration against wild-type SARS-CoV-2 by ELISA in maternal serum and neonatal cord blood.

Case	Vaccine Dose	SRBD IgG by LFI with SR (mothers) (ng/ml)	Nab concentration (wild-type) (mothers) (U/mL)	SRBD IgG by LFI with SR (neonates) (ng/ml)	Nab concentration (wild-type) (neonates) (U/mL)
1	2	200.35	2197.69	154.12	1838.23
2	2	356.83	2316.43	392.56	2334.98
3	2	267.39	2391.26	331.32	2324.96
4	2	76.17	1688.05	107.25	1606.50
5	2	117.91	2310.32	223.79	2289.23
6	2	97.07	2273.42	207.86	2243.51
7	2	138.09	2291.56	158.50	2249.92
8	2	143.27	2292.50	74.03	2235.75
9	1	0.00	2.21	0.00	3.05

10	2	176.04	2364.13	236.61	2366.06
11	2	127.55	1761.85	131.62	2003.60
12	2	110.61	2310.32	178.40	2331.60
13	2	44.14	1488.92	74.42	2099.74
14	2	199.34	2375.24	26.82	2068.32
15	2	320.01	2333.98	134.99	2326.85
16	2	373.35	2340.64	330.32	2367.99
17	2	157.14	2367.02	188.03	2337.31
18	1	0.00	24.30	0.00	30.89
19	1	25.73	149.98	27.84	32.97
20	2	93.46	1927.12	261.04	2226.20

SRBD, spike protein receptor binding domain; ELISA, enzyme-linked immunosorbent assay; LFI, lateral flow immunoassay; SR, spectrum reader; Nab, neutralizing antibody.
